# A tool for assessing continuity of care across care levels: an extended psychometric validation of the CCAENA questionnaire

**DOI:** 10.5334/ijic.1160

**Published:** 2013-12-03

**Authors:** Marta-Beatriz Aller, Ingrid Vargas, Irene Garcia-Subirats, Jordi Coderch, Lluís Colomés, Josep Ramon Llopart, Manel Ferran, Inma Sánchez-Pérez, M. Luisa Vázquez

**Affiliations:** Health Policy and Health Services Research Group, Health Policy Research Unit. Consortium for Health Care and Social Services of Catalonia, Barcelona, Spain; Health Policy and Health Services Research Group, Health Policy Research Unit, Consortium for Health Care and Social Services of Catalonia, Barcelona, Spain; Health Policy and Health Services Research Group, Health Policy Research Unit, Consortium for Health Care and Social Services of Catalonia, Barcelona, Spain; Grup de Recerca en Serveis Sanitaris i Resultats en Salut (GRESSIRES), Serveis de Salut Integrats Baix Empordà, PalamÓs, Spain; Health Policy and Health Services Research Group; Strategic Planning Division, SAGESSA Group, Reus, Spain; Health Policy and Health Services Research Group; Division of Management, Planning and Organizational Development, Badalona Healthcare Services (BSA), Badalona, Spain; Teaching Department, Catalan Health Institute (ICS), Barcelona, Spain; Grup de Recerca en Serveis Sanitaris i Resultats en Salut (GRESSIRES), Serveis de Salut Integrats Baix, PalamÓs, Spain; Health Policy and Health Services Research Group, Health Policy Research Unit, Consortium for Health Care and Social Services of Catalonia, Barcelona, Spain

**Keywords:** continuity of patient care, questionnaires, outcome and process assessment (health care), delivery of health care

## Abstract

**Background:**

The CCAENA questionnaire was developed to assess care continuity across levels from the patients’ perspective. The aim is to provide additional evidence on the psychometric properties of the scales of this questionnaire.

**Methods:**

Cross-sectional study by means of a survey of a random sample of 1500 patients attended in primary and secondary care in three health care areas of the Catalan health care system. Data were collected in 2010 using the CCAENA questionnaire. To assess psychometric properties, an exploratory factor analysis was performed (construct validity) and the item-rest correlations and Cronbach's alpha were calculated (internal consistency). Spearman correlation coefficients were calculated (multidimensionality) and the ability to discriminate between groups was tested.

**Results:**

The factor analysis resulted in 21 items grouped into three factors: patient–primary care provider relationship, patient–secondary care provider relationship and continuity across care levels. Cronbach's alpha indicated good internal consistency (0.97, 0.93, 0.80) and the correlation coefficients indicated that dimensions can be interpreted as separated scales. Scales discriminated patients according to health care area, age and educational level.

**Conclusion:**

The CCAENA questionnaire has proved to be a valid and reliable tool for measuring patients’ perceptions of continuity. Providers and researchers could apply the questionnaire to identify areas for health care improvement.

## Introduction

Continuity of care has been garnering more attention in recent years due to the increase in health care complexity, high specialisation and the involvement of a number of services, as well as an increase in patients with chronic diseases and multiple conditions [1,2]. According to the Reid et al. conceptual framework, continuity of care is defined here as ‘the degree to which patients experience care over time as coherent and linked’ [3,4] and it is the result, from the patients’ perspective, of a combination of adequate access to care, good interpersonal skills, good information flow and uptake between professionals and organisations, and good care coordination between professionals to maintain care consistency [3]. Three types of continuity are identified [3,4]: (1) relational: patients’ perceptions of an ongoing, therapeutic relationship with one or more providers, (2) informational: patients’ perceptions of the availability, use and interpretation of information on past events in order to provide care which is appropriate to their current circumstances, and (3) managerial: patients’ perceptions of receiving different services in a coordinated, complementary and unduplicated way. While relational continuity is related to the continuous caring relationship with professionals, both informational and managerial continuity are related to the perception of interaction among providers [3].

In order to monitor and improve continuity of care, it is important to measure it [5], and its assessment should involve the analysis of relational, informational and managerial continuity of care from the patients’ perspective [3,6,7]. Most available instruments are addressed to specific populations [8], such as patients with diabetes [9,10], cancer [11,12], mental illness [13,14] and patients with an unspecified chronic disease [15] or aimed at users attended to in primary care settings or as inpatients [16,17]. The first generic tool developed to address continuity of care across care levels as perceived by health care users, regardless of morbidity, was the CCAENA© questionnaire (Cuestionario Continuidad Asistencial Entre Niveles de AtenciÓn)[18]. This tool is divided into two sections: the first reconstructs the care trajectory for a specific episode, and the second, which is the object of this paper, consists of Likert scales that measure the patients’ perceptions of the three types of continuity. Two subsequent generic tools were designed to explore patients’ perceptions of continuity of care: the Nijmegen Continuity Questionnaire [19] and the questionnaire elaborated by Haggerty et al. [20]. While the CCAENA questionnaire is focused on the perception of the interaction between providers from different care levels, the other tools also include the perception of interaction of professionals from the same care level. The CCAENA questionnaire allows us to explore patients’ trajectories through the health care services in order to identify aspects of health care supply related to continuity of care where improvements could be made. Furthermore, it is the only available questionnaire of this kind that has been designed in a Spanish-speaking country.

The initial validation of the CCAENA questionnaire indicated that this is a useful instrument to assess continuity of care from the patients’ point of view [18]: face and content validity were high, comprehensibility was considered adequate and the interviewer burden was acceptable [18]. Moreover, items showed an adequate internal structure (construct validity), and scales reached acceptable levels of internal consistency. As a result of this testing some changes were made, such as a modification of the items’ scoring system, the positive formulation of all items and the elimination of redundant items [18]. Recently, the questionnaire was applied in a large survey of 1500 users with the aim of analysing patients’ experiences and perceptions of the three types of continuity of care [21,22]. In this article we present a secondary aim, which is to provide additional evidence on the psychometric properties of the scales of the CCAENA questionnaire.

## Methods

A cross-sectional study was carried out by means of a survey among patients of the Catalonian health care system. The three selected areas were Baix Empordà (rural and semi-urban), the city of Girona (urban) and the Ciutat Vella district of Barcelona (urban). A single provider supplies both primary and secondary care services in Baix Empordà (*Serveis de Salut Integrats del Baix Empordà* - SSIBE; a public entity under private law) and in Girona (*Institut Català de la Salut* - ICS; a public entity under public law). In Ciutat Vella, two entities supply primary care (ICS and *Institut de Prestacions d'Assistència Mèdica al Personal Municipal* - PAMEM) and a different entity provides secondary care (Parc de Salut Mar). The population of 18 years or over served by these organisations in the study areas is 74,144 in Baix Empordà, 83,312 in Girona and 99,093 in Ciutat Vella [23].

### Study population

The study population consisted of patients of 18 years of age or over who had received primary and secondary care in the study areas for the same condition in the three months prior to the survey. Patients who had not been attended to by medical professionals or who could not understand or communicate effectively in Spanish or Catalan were excluded.

The sample size was calculated to analyse the multivariate association model between variables at 95% confidence level, to fulfil the de Moivre theorem of expected frequency higher than five as well as to express the fit and likelihood statistics as a chi-square distribution. The required sample size was estimated to be approximately of 400 patients per health care area. The final sample size was 1500 patients, which is sufficient to analyse the psychometric properties of scales [24–26].

A simple random sample of patients without replacement was selected from a list of patients that fulfilled the inclusion criteria. This list was created from records provided by primary care centres and hospitals of the health care areas. A list of substitutes which included individuals of the same sex and age group was used to replace any refusals.

### Measures

The CCAENA questionnaire was designed to comprehensively assess continuity of care across care levels from the users’ perspective [18]. The tool is divided into two sections: the first reconstructs the care trajectory for a specific condition and identifies the elements of continuity and discontinuity of care experienced in the transition between primary care and secondary care. The second section consists of 29 items conceptually related to the patient–primary care provider relationship (relational continuity; 7 items); patient–secondary care provider relationship (relational continuity; 7 items); transfer of medical information across care levels (informational continuity; 4 items); care coherence across care levels (managerial continuity; 7 items) and accessibility across care levels (managerial continuity, 4 items). Items had four response options, which varied according to the item: (1) strongly agree, agree, disagree and strongly disagree, on items related to relational continuity; and (2) always, often, occasionally and never, on items related to informational and managerial continuity.

To estimate scores, items were rated from 0–3 points (from strongly disagree/never to strongly agree/always). When less than two items were missing per scale and case, the simple imputation method based on the mean score of the item was applied. This method is considered to be adequate due to the high proportion of complete cases [27,28]. The second step consisted of adding the items’ scores and dividing by their highest possible score. Lastly, each continuity score was transformed into a categorical variable with four possible values: very low (≤0.25); low (>0.25 to 0.5); high (>0.5 to 0.75); and very high (>0.75) perception of continuity.

### Data collection

Face-to-face interviews with patients were conducted by trained interviewers, mainly at primary care centres (93.7%), but also at patients’ homes (6.1%) and other places chosen by patients (0.2%). Fieldwork took place between January and May 2010.

### Ethical considerations

The study was conducted in accordance with the current European and Spanish legislation on ethical research [29]. Informed consent was obtained from every interviewee participating in the survey and confidentiality of data was assured by conducting the analysis anonymously. The study protocol was approved by the Ethical Committee for Clinical Research Parc de Salut Mar (2009/3414/I).

### Hypothesis

It was hypothesised that the structure of the scales of the CCAENA questionnaire would reproduce the types of continuity of care defined by Reid et al [3]: relational continuity, informational continuity and managerial continuity. Furthermore, although there is insufficient evidence linking patients perception of continuity of care with organisational and individual characteristics [15,20,30–34], it was expected that scores would differ according to the health care area where patients were attended to and also according to some of their individual characteristics (age, educational level and health status).

### Analyses

#### Item analysis

The item frequency distributions and the rate of missing data for each item were explored. With regard to *construct validity*, an exploratory factor analysis was performed in order to assess whether the clustering of items was as expected (structural validity). The number of retained factors was determined by visual examination of the scree plot and the Kaiser criteria of eigenvalues greater than 1. The analysis was performed with a direct oblimin rotation with Kaiser normalisation, an orthogonal rotation type, because it takes correlations between factors into account. Factor loadings were considered meaningful when they exceeded 0.30 or 0.40 [35].

The *internal consistency* of the scales was analysed by considering the item-rest correlations, i.e., the correlation between an item and the scale that is formed by all other items [36]. The Cronbach's alpha of each scale and the Cronbach's of all but the item concerned were also determined [36]. An alpha value of 0.70 or more was considered satisfactory [25].

#### Scale score analysis

The Spearman correlation coefficient was calculated to assess correlation between scales and gain insight into the *multidimensionality of the instrument*. Correlations of less than 0.70 indicate that the constructed factors can be seen as separated scales [37].

Chi-square tests were used to test the ability to *discriminate between groups of patients* according to the health care area where they were attended to and their individual characteristics (age, educational level, self-rated health status and number of clinical conditions). Extreme groups were contrasted with respect to age (18–35 years vs. over 65 years), educational level (no level of education completed vs. university level) and number of clinical conditions (1 condition vs. >3 conditions).

Statistical analyses were performed using Stata statitical package version 11.

## Results

Of the patients contacted, 77.5% refused to take part in the study. However, there were no statistically significant differences between the final sample and the population of study in terms of sex and age. Information on the sociodemographic or health characteristics of the population of study is not available for comparison.

Over half of the respondents were female (55%), had an education on a primary or secondary level (65.4%) and were born in Spain (78%). About half of the patients (57%) perceived that their health status was either good or very good, and 24% reported to be suffering from just one medical condition (Table 1).

### Item analyses

Most items were highly valued (agree/totally agree; always/often), especially those of the relational continuity scales (Table 2). Missing rates were low, and only four items conceptually related to care coherence across care levels (items 17–18, 20–21) showed non-response rates higher than 5%. Item 20, related to the perception of communication among professionals, was excluded due to its high missing rate (35.7%).

#### Construct validity

To examine the factor structure of the scale, only cases in which patients responded to all items were used (*n* = 1063). Three factors had eigenvalues greater than 1 (7.72, 3.59, 1.53) and thus satisfied Kaiser's criterion (Table 3).

Items associated with relational continuity loaded strongly on the first (patient–primary care provider relationship) and second (patient–secondary care provider relationship) factors. The four items related to transfer of information across care levels (items 15–18) and three items related to care coherence across care levels (items 19, 21 and 25) loaded on the third factor, referred to as ‘continuity across care levels’. Items 22–24 and 26–29 did not load on any factor and were excluded from the scales.

#### Internal consistence

Item-rest correlations were higher than 0.4 for all selected items except items 19 and 21 (0.360) and lower than 0.3 for excluded items (Table 3). Taking all items into consideration, the Cronbach's alpha values were 0.936, 0.931 and 0.728 for the patient–primary care provider relationship, patient–secondary care provider relationship and continuity across care levels, respectively. The Cronbach's alpha for the third scale when items 22–24 were eliminated increased to 0.805.

Two different conceptual subscales compose the scale of continuity across care levels: the transfer of medical information subscale (items 15–18) and the care coherence subscale (items 19, 21 and 25). Their associated Cronbach's alpha values after eliminating items 22–24 were 0.766 and 0.635, respectively.

### Scale score analysis

Three scores were calculated: (1) patient–primary care provider relationship - relational continuity (items 1–7), (2) patient–secondary care provider relationship - relational continuity (items 8–14), (3) continuity across care levels - informational and managerial continuity (items 15–19, 21 and 25). Two additional scores were calculated from the scale of continuity across care levels: the transfer of medical information across care levels subscale (items 15–18) and the care coherence across care levels subscale (items 19, 21 and 25).

#### Multidimensionality

Spearman correlation coefficients between scales were lower than 0.70 (Table 4). Subscales ‘transfer of medical information across care levels’ and ‘care coherence across care levels’ showed a correlation coefficient of 0.511, indicating that they can be interpreted as separated scores.

#### Discriminant validity

Table 5 shows the percentage of patients perceiving high or very high continuity of care for each group and the statistical significance testing (*p*-value) of differences in scores between selected subgroups. Scales discriminated patients according to the health care area where patients were attended to, age and level of education. For example, 58.9% of patients aged 18–35 years perceived high or very high levels of continuity across care levels. This percentage reached 89.3% in patients older than 65 years.

Only the scale of continuity across care levels and its subscale of transfer of medical information across care levels were able to discriminate between patients according to their self-rated health status and the number of clinical conditions.

## Discussion

The CCAENA© questionnaire is a generic tool aimed firstly at exploring patients’ trajectories in health care services to identify the elements of (dis)continuity experienced in the transition across care levels [21] and secondly at measuring the degree of continuity of care perceived by users using Likert scales [22]. The questionnaire was previously validated using an expert group, two pretests and a pilot test with a sample of 200 health care users [18]. The results presented are an extended validation of the scales of the questionnaire. Two hypotheses related to the psychometric characteristics of the CCAENA questionnaire and their ability to discriminate between groups were tested in a large sample of patients from different age groups, suffering from diverse medical conditions and attended to in different care settings of the Catalan health care system.

The first hypothesis, i.e. that the structure of the scales would reproduce the classification of the types of continuity of care defined by Reid et al. [3], is partially supported by the factorial analysis, which identified two factors that represented relational continuity (patient–primary and secondary care provider relationship) and one factor representing the perception of continuity across care levels (transfer of medical information across care levels and care coherence across care levels). These scales also demonstrated an adequate level of internal consistency and the multidimensionality of the scales was supported by the inter-factor correlation. The fact that items composing the ‘continuity across care levels’ scale, which are related to informational and managerial continuity, load on the same factor has a theoretical foundation in recent studies which argue that the most relevant distinction of patients is between relational continuity and ‘seamless care’, which includes aspects of both informational and managerial continuity of care [5,38]. In the validation process of the Nijmegen Continuity Questionnaire, a similar structural distribution of items was observed [19]. We also explored the psychometric properties of the two subscales that at a conceptual level compose the ‘continuity across care levels’ scale: the ‘transfer of medical information across care levels’ and ‘care coherence across care levels’. This subdivision is supported by the conceptual framework that guided the study [3] and by the reliability and multidimensionality analyses, which demonstrate that the internal consistency of each scale is adequate and the scales of informational and managerial continuity are sufficiently uncorrelated to consider them separately.

The second hypothesis, i.e. that the scores would be able to differentiate perceptions between patient groups, is partially supported by the discriminant analyses: scales discriminate patients according to the health care area where they were attended to and according to some of their individual characteristics - i.e. sex and age. Only the ‘continuity across care levels’ scale and its ‘transfer of medical information across care levels’ subscale were able to discriminate patients according to their health status. However, evidence linking continuity of care and morbidity is inconclusive [15,30,34]. Therefore there are two possible explanations: on the one hand, that there are differences in relational and care coherence perceptions according to the health status of patients but scales lack sufficient discriminant ability to detect those differences; on the other hand, it is also possible that perceptions of these types of continuity do not vary according to health status, as suggested by some research [15,30]. This issue should be further explored in future research.

The test suggested that it was advisable to remove four items related to care coherence and four items related to accessibility across care. However, the information provided by these items is highly relevant to our understanding of patients’ continuity of care perceptions and they have been maintained in the questionnaire. The structural behaviour of items related to care coherence could be explained by the fact that its theoretical construct is composed of a number of attributes that are not necessarily correlated among themselves as previously observed [18], i.e. perception of care coordination, communication between services, no duplication of tests or exams, adequate sequence of health care and appropriate follow-up of patients. The lack of internal consistency of items related to accessibility across care levels could be explained by the fact that accessibility to primary care and accessibility to secondary care do not necessarily need to be correlated. The possibility of splitting the scale into two different scales (items 26 and 29; items 27–28) was rejected because the literature recommends that scales have at least three items [39].

In conclusion, this research is in line with those of the previous validation process [18], and suggests that the CCAENA questionnaire is an adequate tool for measuring patients’ perceptions of continuity of care. Providers and researchers interested in improving continuity of care across care levels could apply the CCAENA questionnaire to identify areas for improvement.

## Figures and Tables

**Table 1.**
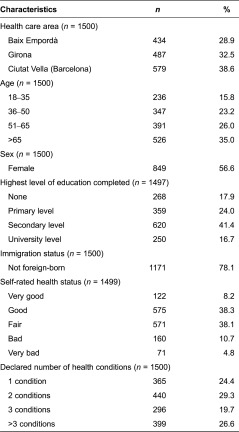
Characteristics of the study sample

**Table 2.**
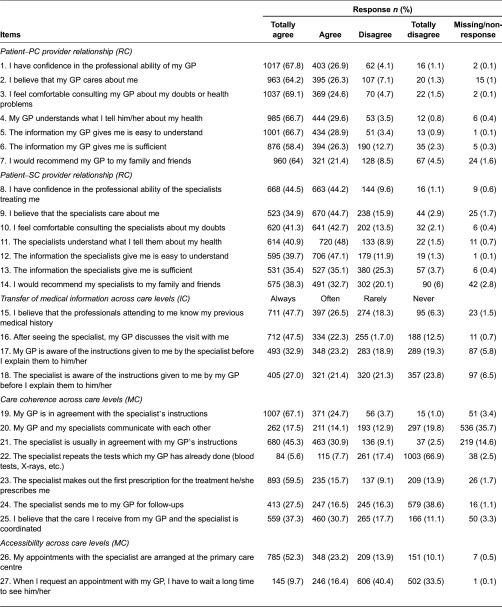

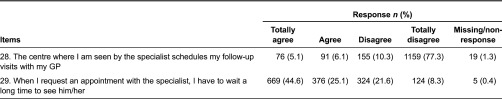
Items response distribution and missing data

IC, informational continuity; MC, managerial continuity; RC, relational continuity.

**Table 3.**
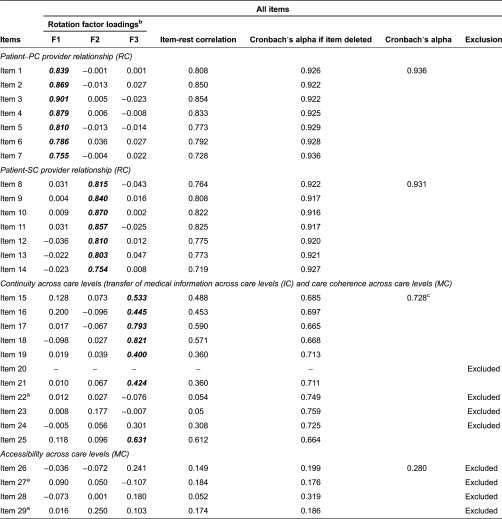
Factor analysis, item-rest correlation and Cronbach's alpha of the CCAENA items

^a^Response values have been reversed for analysis.

^b^Eigenvalues and percentage of variance accounted for by factors I, II and III were 7.725, 3.593 and 1.529, respectively.

^c^Cronbach's alpha of the definitive scale (items 15–19, 21 and 25) is 0.805.

IC, informational continuity; MC, managerial continuity; RC, relational continuity. Factor loadings that exceded 0.400 are shown in bold.

**Table 4.**
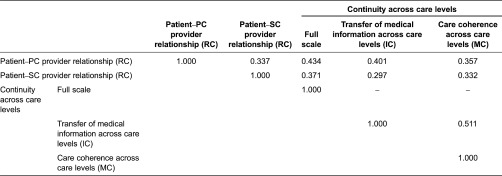
Spearman correlation coefficients between scales^a^

IC, informational continuity; MC, managerial continuity; PC, primary care; RC, relational continuity; SC, secondary care.

^a^Spearman correlation coefficients calculated using definitive scales.

**Table 5.**
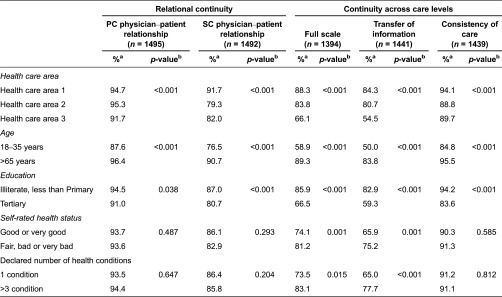
Differences in scores between subgroups

^a^Percentage of patients perceiving high or very high continuity of care.

^b^*P*-value associated for Chi-square test comparing very high, high, low and very low perceptions of continuity of care among groups.

IC, informational continuity; MC, managerial continuity; PC, primary care; RC, relational continuity; SC, secondary care.
